# ICIS workshop on adnexal mass characterization

**DOI:** 10.1186/1470-7330-14-S1-O17

**Published:** 2014-10-09

**Authors:** Andrea Rockall

**Affiliations:** 1 Imperial College Healthcase NSH Trust, UK

## 

Ovarian cancer is predominantly diagnosed at a late stage due to a relative lack of symptoms in the early stages of disease. The published screening trials have not demonstrated any benefit to screening for disease. However, with relatively widespread use of pelvic ultrasound for abdominal or pelvic symptoms, adnexal masses are commonly identified. The majority of such adnexal masses are benign and the majority can be readily triaged into benign or malignant categories using standardized systems, such as the IOTA simple rules. However, a number of cases are difficult to categorise and remain indeterminate on ultrasound. It is important to categorise each lesion as accurately as possible to direct the appropriate management, ensuring cytoreductive surgery in cases that are highly likely to be cancer whilst allowing a more conservative approach in benign cases, particularly when fertility preservation is of concern to the patient. There is a body of evidence now to support the role of MRI in the assessment of this cohort of “difficult to classify” adnexal masses. The T2 signal intensity characteristics and the presence of enhancement are important classifiers. Very low T2 signal intensity and lack of enhancement are markers of benignity. More recently, the role of diffusion weighted imaging (DWI) and dynamic contrast enhancement (DCE) has been closely evaluated. The enhancement curves generated from DCE can be a strong pointer to benignity or malignancy, using the myometrial enhancement as an internal comparator. Where the solid component enhances more rapidly and more intensely than the myometrium, invasive malignancy is highly likely. In lesions that retain no high signal intensity on the high b value DWI, benignity is highly likely. A proposed scoring system, the AdnexMR score, has been developed and is currently being prospectively tested throughout Europe and several centres in the United States. This workshop will review the key features for characterizing adnexal masses, review the proposed scoring system, and give practice examples for scoring by the audience.

